# Characterization of plasmids carrying *bla*_CTX-M_ genes among extra-intestinal *Escherichia coli* clinical isolates in Ethiopia

**DOI:** 10.1038/s41598-023-35402-2

**Published:** 2023-05-26

**Authors:** Abebe Aseffa Negeri, Hassen Mamo, Dharmender K. Gahlot, Jyoti M. Gurung, Eyasu Tigabu Seyoum, Matthew S. Francis

**Affiliations:** 1 National Clinical Bacteriology and Mycology Reference Laboratory, Ethiopian Public Health Institute, Addis Ababa, Ethiopia; 2 Department of Microbial, Cellular and Molecular Biology, College of Natural and Computational Sciences, Addis Ababa University, Addis Ababa, Ethiopia; 3 Department of Molecular Biology, Umeå University, Umeå, Sweden; 4 Umeå Centre for Microbial Research, Umeå University, Umeå, Sweden; 5 Global One Health Initiative of the Ohio State University, East African Regional Office, Addis Ababa, Ethiopia

**Keywords:** Antimicrobial resistance, Infection, Applied microbiology, Policy and public health in microbiology

## Abstract

CTX-Ms are encoded by *bla*_CTX-M_ genes and are widely distributed extended-spectrum β-lactamases (ESBLs). They are the most important antimicrobial resistance (AMR) mechanism to β-lactam antibiotics in the Enterobacteriaceae. However, the role of transmissible AMR plasmids in the dissemination of *bla*_CTX-M_ genes has scarcely been studied in Africa where the burden of AMR is high and rapidly spreading. In this study, AMR plasmid transmissibility, replicon types and addiction systems were analysed in CTX-M-producing *Escherichia coli* clinical isolates in Ethiopia with a goal to provide molecular insight into mechanisms underlying such high prevalence and rapid dissemination. Of 100 CTX-Ms-producing isolates obtained from urine (84), pus (10) and blood (6) from four geographically distinct healthcare settings, 75% carried transmissible plasmids encoding for CTX-Ms, with CTX-M-15 being predominant (n = 51). Single IncF plasmids with the combination of F-FIA-FIB (n = 17) carried the bulk of *bla*_CTX-M-15_ genes. In addition, IncF plasmids were associated with multiple addiction systems, IS*Ecp*1 and various resistance phenotypes for non-cephalosporin antibiotics. Moreover, IncF plasmid carriage is associated with the international pandemic *E. coli* ST131 lineage. Furthermore, several CTX-M encoding plasmids were associated with serum survival of the strains, but less so with biofilm formation. Hence, both horizontal gene transfer and clonal expansion may contribute to the rapid and widespread distribution of *bla*_CTX-M_ genes among *E. coli* populations in Ethiopian clinical settings. This information is relevant for local epidemiology and surveillance, but also for global understanding of the successful dissemination of AMR gene carrying plasmids.

## Introduction

Antimicrobial resistance (AMR) is an acute problem of the global healthcare system^1^. The issue is typically associated with the extensive Enterobacteriaceae family^2^. In this bacterial family, extended-spectrum β-lactamases (ESBLs) are the primary AMR mechanism nullifying β-lactam type antibiotics. CTX-Ms encoded by the *bla*_CTX-M_ genes are the most dominant and widespread ESBL enzyme types, with *Escherichia coli* being a major source of their production^3,4^. Tracking of CTX-Ms-producing *E. coli* is challenging, and requires in-depth investigation of the underlying dissemination mechanisms, clinical implications, and overall epidemiology of the *bla*_CTX-M_ genes.

Bacterial plasmids are vital mobile elements for horizontal transfer between different bacterial species of exogenous genes including AMR genes. This has been extensively investigated in high-income countries^5,6^. The incompatibility (Inc) group F (IncF) which belongs to the narrow-host-range plasmids is the most relevant to AMR gene transmission^7^. Horizontal spread of AMR plasmids also plays an important role in the acquisition of virulence encoding genes^8^. For instance, ESBLs-encoding plasmids are associated with enhanced virulence potential in pandemic *E. coli* sequence type (ST) 131 strain and other pathogenic *E. coli*^9^.

In Africa, despite the high burden of ESBL-producing *E. coli*^10^, there exists little knowledge of the underlying molecular mechanisms, virulence associated factors and overall plasmid-mediated AMR epidemiology. Without unambiguous and up-to-date data on this, effective tracking and management of virulent *E. coli* is not feasible. We have recently published a phenotypic characterization of CTX-M-positive *E. coli* clinical isolates from four geographically distinct healthcare settings in Ethiopia^11^. The study reported on the different *E. coli* phylo-groups with several isolates belonging to the international high risk ST131 clone^11^. It also revealed the presence of alternative β-lactamase gene carriage in certain isolates and obvious multidrug resistance (MDR) among them^11^. It is of interest to know if these characteristics are transferrable to recipient *E. coli* strains through transmissible AMR plasmids.

To limit the effectiveness of plasmid transmissibility, and thereby the spread of MDR, it is first necessary to understand the biology of the existing AMR plasmids. To our knowledge, this has not been conducted on plasmids identified in clinical *E. coli* isolates from Ethiopia. Hence, in this study we began this important work by focusing first on transferability of AMR plasmids and identifying the plasmid replicon types among the clinical isolates used in our previous study. This study also investigated the association between the IS*Ecp1* element and *bla*_CTX-M_ genes because this insertion system element is commonly found adjacent to the CTX-M encoding genes on transmissible plasmids, and plays an important role in chromosomal capture, mobilization, and expression of the AMR genes from the transmissible plasmids^6,7,12^. We also assessed the presence of eight plasmid encoded addiction systems since plasmid maintenance during host replication is a vital aspect of transmissibility^13,14^. Finally, because plasmid replication, maintenance, and transfer are influenced by bacteria living in biofilm communities^15^ and that AMR plasmid carriage impacts on serum resistance^16^, an important virulence property of pathogenic *E. coli* strains^17^, these associations were also investigated in the current study. By doing so, this study is the first to describe the biology of the existing AMR plasmids among clinical *E. coli* isolates from Ethiopia and can therefore contribute important knowledge of processes leading to the rapid dissemination of MDR, and that can then be identified as targets for inhibition.

## Results

### AMR plasmid transmissibility

To determine the molecular basis for the spread of CTX-M genes, we semi-randomly selected 100 CTX-M producing isolates from the original study^11^ (Supplementary Table S1). The basis for study inclusion were the criteria (1) all isolates must be CTX-M producing, (2) all geographical study sites must be represented [NRL (National reference laboratory)—36; TASH (Tikur Anbessa Specialized hospital)—31; ARH (Ayder Referral Hospital) 19; JUH (Jimma University Hospital)—14], and (3) all phylogenetic groups must be represented [phylogroup A (19); B1 (4); B2 (52); C (16); D (5), and F (4)] (Table 1). We were able to demonstrate that 75% (n = 75) of these clinical isolates had the ability to transfer genes encoding CTX-M determinants to *E. coli* J53 AziR by conjugation or to *E. coli* HB101 by chemical transformation (Table 1). Of the 75 isolates that demonstrated transmissibility, the gene encoding for CTX-M-15 was represented in 51 isolates (68.0%), other group-1 CTX-M genes were in 17 isolates (22.7%), and group 9 CTX-M genes in 7 isolates (9.3%) (Table 1). Of the 25% (n = 25) of clinical isolates where transmissibility could not be demonstrated, we identified CTX-M encoding genes among five phylo-groups, and the *bla*_CTX-M-15_ gene in B2-ST131 isolates was dominant (Table 1). Sequencing of the amplified DNA fragments confirmed an association between the IS*Ecp1* and *bla*_CTX-M_ genes. Strikingly, 73 of the 75 parental isolates (97.3%) and 71 (94.7%) of the recipient strains were positive for the IS*Ecp1* element*.*

**Table 1:** The transmissibility of 100 CTX-M producing clinical *E. coli* isolates included in this study.

Phylogenetic groups^a^	Transmissible (n = 75)			Non-transmissible (n = 25)		
	CTX-M-15	Other CTX-M group 1	CTX-M group 9	CTX-M-15	Other CTX-M group 1	CTX-M group 9
A (19)	11	2	1	4	1	–
B1 (4)	2	2	–	–	–	–
B2 (52)^b^	30 *(28)*	6 *(6)*	6 *(6)*	8 *(8)*	1	1 (*1*)
C (16)	6	2	–	6	1	1
D (5)	1	3	–	1	–	–
F (4)	1	2	–	1	–	–
Total^c^	51	17	7	20	3	2

^a^Determination of the distribution of the isolates among the six phylogenetic groups was performed in our previous study^11^. Numbers in parentheses indicate the number of isolates belonging to a phylogroup that were selected for use in this study. The geographical origin of these strains can be identified from information provided in supplementary Table S1.

^b^Numbers in parentheses that are italicised indicate B2 isolates of international high-risk clone ST131.

^c^The degree of transmissibility of *bla*_CTX-M-15_, other group 1 *bla*_CTX-Ms_, and group 9 *bla*_CTX-Ms_ is 68% (51 of 75), 22.7% (17/75) and 9.3% (7/75), respectively.

Alternative β-lactamase-encoding genes (non-ESBLs genes) were detected in 72 of the 75 isolates that could transmit AMR genes to recipient bacteria. Specifically, *bla*_TEM-1_, *bla*_OXA-1_ and *bla*_TEM-OXA_ genes were detected in 13 (17.3%), 16 (21.3%) and 39 (52%) of the 75 original parental isolates respectively (Table 2). The ESBL *bla*_SHV_ gene was detected in 4 (5.3%) isolates. Three isolates (4%) were devoid of all four of these alternative β-lactamase-encoding genes. These same genotypes were characterized in the plasmid recipient strains. The *bla*_TEM-1_, *bla*_OXA-1_ and *bla*_TEM-OXA_ genes were detected in 23 (30.7%), 13 (17.3%) and 26 (34.7%) of the recipient strains receiving mobilised AMR plasmid(s), respectively (Table 2). The remaining 13 (17.3%) recipient strains did not acquire any of these genes. Furthermore, not a single recipient strain gained the ESBL *bla*_SHV_ gene (Table 2).

**Table 2:** The distribution of alternative β-lactamases among CTX-M producing clinical *E. coli* isolates and recipient strains.

Phylogenetic groups^a^	Parental strains (n = 75)					Recipient strains (n = 75)				
	TEM	OXA	TEM-OXA	SHV	Negative	TEM	OXA	TEM-OXA	SHV	Negative
A	4	3	4	1	2	3	3	3	0	4
B1	2	0	2	0	0	2	0	2	0	0
B2	6	12	21	3	0	14	8	12	0	6
C	0	1	9	0	1	3	1	7	0	0
D	1	0	3	0	0	0	1	1	0	2
F	0	0	3	0	0	1	0	1	0	1
Total^b^	13	16	39	4	3	23	13	26	0	13

^a^Determination of the distribution of the isolates among the six phylogenetic groups is presented as in Table 1 and derived from our previous study^11^.

^b^Observe that the total number of parental strains positive for TEM (n = 13) and TEM-OXA (n = 39) is 52. The number of recipient strains positive for TEM (n = 23) and TEM-OXA (n = 26) is 49. This means that the number of single TEM positive recipient strains increase to 23 from the original 13 parental strains that were positive. On the other hand, the number of TEM-OXA positive recipient strains decrease to 26 from the original 39 parental strains that were positive. Hence, some parental strains transferred TEM only rather than TEM and OXA together.

### Acquisition of MDR

**Figure 1:**
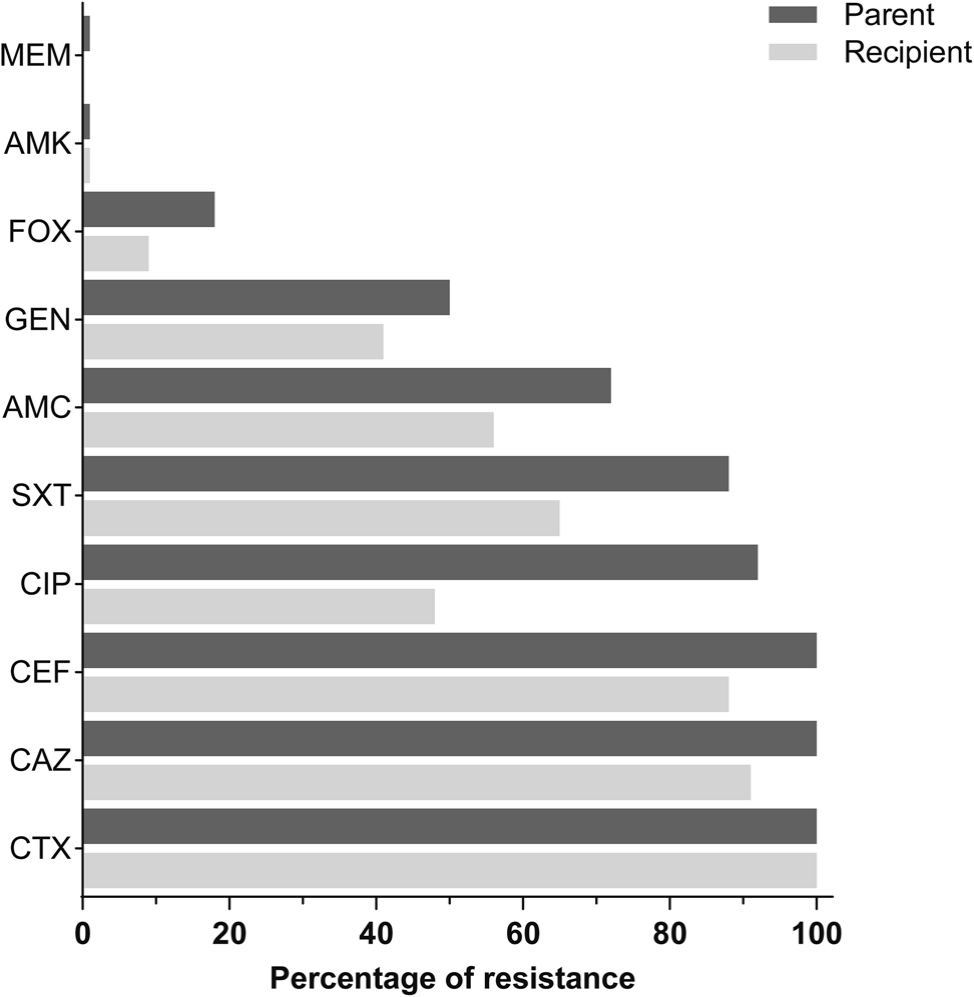
Comparison of the percent antibiotic resistance displayed by 75 original isolates and the corresponding plasmid recipient strains. Recipient strains are based upon either the *E. coli* J53 AziR background and achieved via conjugal mating (transconjugates) or the *E. coli* HB101 background achieved via chemical transformation (transformants). The percent resistance of the different parent (dark grey bars) and recipient (light grey bars) isolates was according to the CLSI disk diffusion breakpoints. Resistance was defined as isolates with intermediate resistance and complete resistance based upon the size of the inhibition zone compared to the reference strains ESBL negative *E. coli* ATCC 25,922 and ESBL positive *K. pneumoniae* subsp. pneumoniae ATCC 700,603. Antibiotics tested were amoxicillin-clavulanate (AMC), cefotaxime (CTX), ceftazidime (CAZ),), cefepime (CEF), cefoxitine (FOX), ciprofloxacin (CIP), Amikacin (AMK), gentamicin (GEN), Meropenem (MEM), Sulphamethoxazole-Trimethoprim (SXT).

We had previously assessed multiple drug resistance profiles among the parental clinical isolates used in this study^11^ (Supplementary Table S1). To address whether this characteristic could be conferred to recipient bacteria through the transmissibility of AMR plasmids, we performed an antimicrobial susceptibility test using the disk diffusion method on all 75 parental clinical isolates and their corresponding recipients of AMR plasmids. The 75 isolates were 100% resistant to cefotaxime, ceftazidime and cefepime confirming our earlier finding^11^. Among second-generation plasmid recipient strains this resistance rate remained 100% for cefotaxime and dropped slightly to 90.7% and 88.0% for ceftazidime and cefepime respectively (Fig. 1). The resistance rates for ciprofloxacin, sulfamethoxazole/trimethoprim amoxicillin-clavulanic acid, gentamicin, cefoxitin, amikacin and meropenem were 92.0%, 88.0%, 72.0%, 50.7%, 17.3%, 1.3% and 1.3%, respectively (Fig. 1). Among the AMR plasmid recipient strains, resistance rates to these non-β-lactams antibiotics were 48%, 65.3%, 41.3% and 1.3% for ciprofloxacin, sulfamethoxazole/trimethoprim, gentamicin, and amikacin respectively (Fig. 1). Moreover, 56% of the second-generation strains were resistant to the β-lactamase inhibitor amoxicillin-clavulanic acid and 9.3% were resistant to cefoxitin, whereas no strain acquired resistance to meropenem (Fig. 1).

### AMR plasmid replicon types

**Figure 2:**
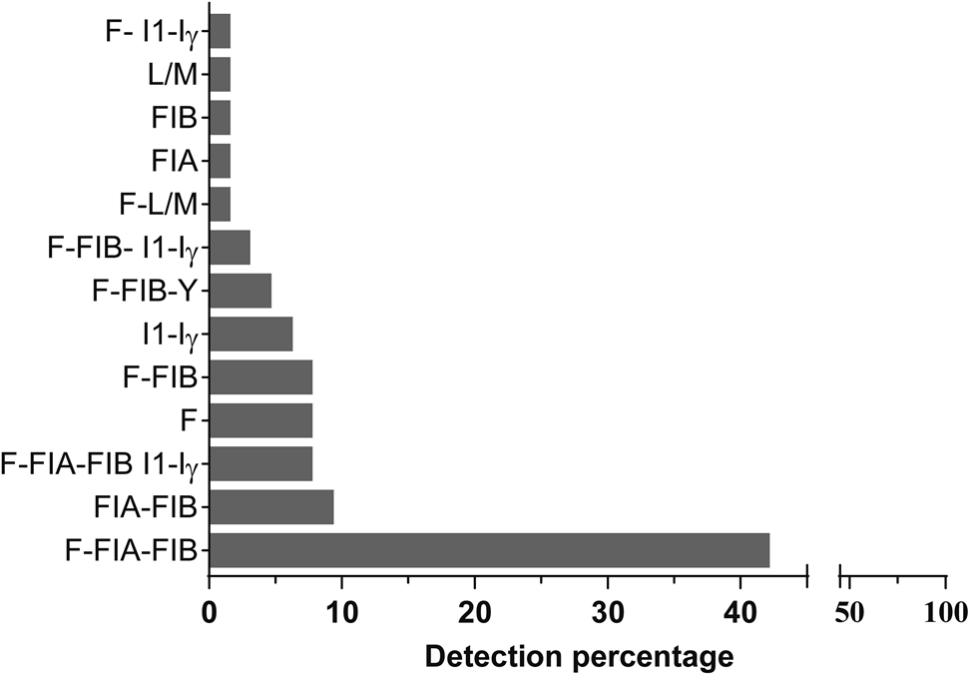
Frequency of plasmid replicon types originated from *E. coli* isolates obtained from clinical samples collected from healthcare centers in Ethiopia. Of 75 recipient strains receiving one or more plasmids via conjugation or transformation, 64 contained plasmids that could be typed by the chosen PCR-based method. IncF based replicon types were most identified.

To our knowledge, the biology of AMR plasmids harboured by clinical bacterial isolates from Ethiopia has not been studied. We began this important work by focusing first on identifying the plasmid replicon types among the isolates used in our study by adopting an established PCR based replicon typing (PBRT) protocol^18^. Of the 75 transferred CTX-M ESBLs-encoding plasmids, replicons of 64 (85.3%) were typed and revealed four Inc groups categorized into 13 different combinations (Fig. 2). The IncF plasmids with various replicon types were the most numerous combinations among all the plasmids. The plasmid F-FIA-FIB replicon groups were the most frequently identified (42.2%) with 27 of the 64, followed by the combination of FIA-FIB (9.4%) (Fig. 2). The replicon types F-FIB-I1-Iγ, F-FIB as well as IncF replicon types were all detected at a frequency of 7.8%, and the I1-Iγ replicon type at 6.3% (Fig. 2). The other types identified were detected at below 5% frequency in the plasmid recipient strains (Fig. 2).

We also determined the association of replicon types with antibiotic resistance genes. Out of the 51 transmissible plasmids having *bla*_CTX-M-15_ (see Table 1), 45 (88.2%) were typed using PBRT. Among these 45 PBRT-typed transmissible plasmids, 35 (77.8%) carried combinations of Inc replicon types while the remaining 10 (22.2%) carried just a single replicon type (Table 3). The combination of F-FIA-FIB plasmid replicon was frequently associated with other group-1 CTX-M types encoded by *bla*_CTX-M-101_, *bla*_CTX-M-103_, *bla*_CTX-M-142_, *bla*_CTX-M-180_, *bla*_CTX-M-182_ and *bla*_CTX-M-225_ (Table 3). In addition, 6 plasmids carrying group-9 *bla*_CTX-M-27_ belonged to 3 different replicon types consisting of a single IncF (n = 1), as well as in apparent combination with F-FIA-FIB (n = 4), or with F-FIA-FIB, and I1-Iγ (n = 1) (Table 3).

**Table 3:** Distribution and number of plasmid replicon types and associated addiction systems in recipient *E. coli* containing transmissible plasmids categorised according to CTX-M carriage and plasmid replicon type.

Plasmid replicon type	n	Number of isolates with identified addiction system							
		*pemKI*	*ccdAB*	*vagCD*	*hok-sok*	*pndAC*	*srnBC*	total^a^	mean^b^
CTX-M-15 plasmids	51	33	28	15	2	17	28	123	2.4
F	3	1	0	0	0	1	1	3	1.0
F, FIA, FIB	17	15	14	10	1	6	17	63	3.7
F, FIA, FIB, I1-Iγ	3	3	3	2	1	2	3	14	4.7
F, FIB, I1-Iγ	2	0	0	0	0	2	1	3	1.5
F, FIB, Y	3	3	3	0	0	2	0	8	2.7
F, FIB	5	4	4	0	0	0	3	11	2.2
F, L/M	1	1	1	0	0	0	0	2	2.0
FIA, FIB	4	1	0	1	0	1	1	4	1.0
FIA	1	0	1	0	0	0	0	1	1.0
FIB	1	1	1	0	0	0	0	2	2.0
I1-Iγ	4	1	0	1	0	3	1	6	1.5
L/M	1	0	0	0	0	0	0	0	0.0
Not identified	6	3	1	1	0	0	1	6	1.0
Others group 1 CTX-M	17	10	6	3	1	3	9	32	1.9
F	1	1	0	0	0	0	0	1	1.0
F, FIA, FIB	6	5	3	2	0	1	6	17	2.0
F, FIA, FIB, I1-Iγ	1	1	1	0	1	0	1	4	4.0
F, I1-Iγ	1	0	0	0	0	1	0	1	1.0
FIA, FIB	2	1	1	1	0	0	1	4	2.0
FIA,FIB, I1-Iγ	2	2	1	0	0	1	1	5	2.5
Not identified	4	0	0	0	0	0	0	0	0.0
CTX-M-14 plasmid	1	0	0	0	0	0	0	0	0.0
Not identified	1	0	0	0	0	0	0	0	0.0
CTX-M-27 plasmids	6	4	5	3	1	3	5	21	3.0
F	1	0	0	0	0	1	0	1	1.0
F, FIA, FIB	4	3	4	2	0	1	4	14	3.5
F, FIA, FIB, I1-Iγ	1	1	1	1	1	1	1	6	6.0
Total	75	47	39	21	4	23	42	176	2.3

^a^Refers to the total number of isolates that had an identifiable addiction system encoded on a plasmid with defined replicon type.

^b^Refers to the average number of addiction systems encoded on a plasmid with defined replicon type; calculated as ‘total’ number of identified addiction systems divided by ‘n’ number of isolates containing a plasmid(s) with stated replicon type.

In contrast, the PBRT protocol was unable to type into any of the 18 incompatibility groups the remaining 11 (14.7%) of 75 isolates containing transmissible plasmids carrying *bla*_CTX-M_ genes. Of these untyped 11 isolates, 6 (54.5%) contained a plasmid carrying *bla*_CTX-M-15_, 4 (36.4%) had a plasmid having genes encoding for other group-1 CTX-Ms (1 *bla*_CTX-M-55_, 2 *bla*_CTX-M-180_, 1 *bla*_CTX-M-182_) and 1 (9.1%) with plasmid carrying group-9 *bla*_CTX-M-14_ (Table 3).

### Addiction systems for plasmid maintenance

Plasmid maintenance during host replication is a vital aspect of transmissibility. We investigated the presence of eight plasmid encoded addiction systems according to a previously described PCR-based detection system^19^. Six plasmid addiction system types (*pemKI*, *ccdAB*, *vagCD*, *hok-sok*, *pndAC* and *srnBC*) could be identified among the 75 parental isolates (Table 4) and the corresponding plasmid recipient strains (Table 5). In the parental strains, the 337 plasmid addition system combinations detected were *pemKI* (n = 72), *srnBC* (n = 68), *ccdAB* (n = 68), *vagCD* (n = 55), *pndAC* (n = 48), and *hok-sok* (n = 26). The *relBE* and *parDE* plasmid addiction systems were not detected in the parental *E. coli* strains analysed. On the other hand, in the plasmid recipient strains, a total of 176 plasmid addiction system combinations were identified consisting of *pemKI* (n = 47), *srnBC* (n = 42), *ccdAB* (n = 39), *vagCD* (n = 21), *pndAC* (n = 23), and *hok-sok* (n = 4). Once again, none of the strains harboured the *relBE* and *parDE* plasmid addiction systems.

**Table 4:** Distribution of addiction systems among transmissible plasmids encoding CTX-M genes and harboured by donor parental strains of clinical origin in Ethiopia.

CTX-M type ESBL with other β-lactamases	n	Addiction systems							
		*pmeK*	*ccdAB*	*vagCD*	*hok-sok*	*pndAC*	*srnBC*	total^a^	mean^b^
CTX-M-15	51	50	49	40	18	33	46	237	4.6
only CTX-M-15	2	2	2	2	0	1	2	9	4.5
+ TEM-1	7	7	7	6	3	6	6	35	5.0
+ OXA-1	12	13	12	11	6	9	12	63	4.8
+ TEM-1, OXA-1	29	28	28	20	9	17	26	128	4.4
Other group 1 CTX-Ms	17	16	14	11	6	10	16	73	4.3
+ TEM-1	4	3	3	1	0	2	4	13	3.3
+ OXA-1	4	4	3	4	2	3	4	20	5.0
+ TEM-1, OXA-1	9	9	8	6	4	5	8	40	4.4
Group 9 CTX-Ms	7	6	5	4	2	5	6	28	4.0
CTX-M-14 + TEM-1	1	0	0	0	0	0	0	0	0
only CTX-M-27	1	1	1	1	1	1	1	6	6.0
+ TEM-1	1	1	1	1	1	1	1	6	6.0
+ TEM, OXA-1	4	4	3	2	0	3	4	16	4.0
Total	75	72	68	55	26	48	68	337	4.5

^a^Refers to the total number of isolates that had an identifiable addiction system encoded on a plasmid with defined CTX-M producing genes alone or in combination with alternative β-lactamase-encoding genes (non-ESBLs genes).

^b^Refers to the average number of addiction systems encoded on a plasmid with defined β-lactamase-encoding genes; calculated as ‘total’ number of identified addiction systems divided by ‘n’ number of isolates containing a plasmid(s) with stated β-lactamase-encoding gene.

**Table 5:** Distribution of addiction systems among CTX-M-encoding transmissible plasmids that were successfully mobilised to recipient *E. coli*^a^*.*

CTX-M with other β-lactamases	n	Plasmids Addiction systems							
		*pmeK*	*ccdB*	*vagCD*	*hok-sok*	*pndC*	*srnBC*	total^b^	Mean^c^
CTX-M-15	51	33	28	15	2	17	28	123	2.4
Only CTX-M-15	5	3	3	1	0	2	3	12	2.4
+ TEM-1	19	7	5	0	0	7	4	23	1.3
+ OXA-1	9	9	9	6	1	3	9	37	4.1
+ TEM, OXA-1	18	14	11	8	1	5	12	51	2.8
Other group-1 CTX-Ms	17	10	6	3	1	3	9	32	1.9
Only CTX-M (other)	5	2	2	2	0	1	2	9	1.8
+ TEM	3	1	1	0	0	2	1	5	1.7
+ OXA-1	3	3	1	1	0	0	3	8	2.7
+ TEM, OXA-1	6	4	2	0	1	0	3	10	1.7
Group 9 CTX-M	7	4	5	3	1	3	5	21	3.0
CTX-M14 + TEM	1	0	0	0	0	0	0	0	0
CTX-M-27	3	2	2	1	1	2	2	10	3.3
+ OXA-1	1	1	1	0	0	0	1	3	3.0
+ TEM, OXA-1	2	1	2	2	0	1	2	8	4.0
Total	75	47	39	21	4	23	42	176	2.3

^a^Mobilisation of transmissible plasmids into *E. coli* J53 AziR was by conjugation and into HB101 was by chemical transformation.

^b^Refers to the total number of isolates that had an identifiable addiction system encoded on a plasmid with defined CTX-M producing genes alone or in combination with alternative β-lactamase-encoding genes (non-ESBLs genes).

^c^Refers to the average number of addiction systems encoded on a plasmid with defined β-lactamase-encoding genes; calculated as ‘total’ number of identified addiction systems divided by ‘n’ number of isolates containing a plasmid(s) with stated β-lactamase-encoding gene.

There was direct correlation between the combination of plasmid replicon types and the mean numbers of addiction systems detected (Table 3). The highest mean numbers of addiction systems were observed in plasmids with a combination of four replicon types. In contrast, the lowest mean numbers of addiction systems correlated to plasmids with a single Inc replicon type. However, no clear correlation between any of the mean numbers of plasmid addiction system combinations and any β-lactamase type including the CTX-M in either the donor parental strains (Table 4) or the recipient strains (Table 5) could be identified.

### Plasmid transmissibility and biofilm formation

**Figure 3:**
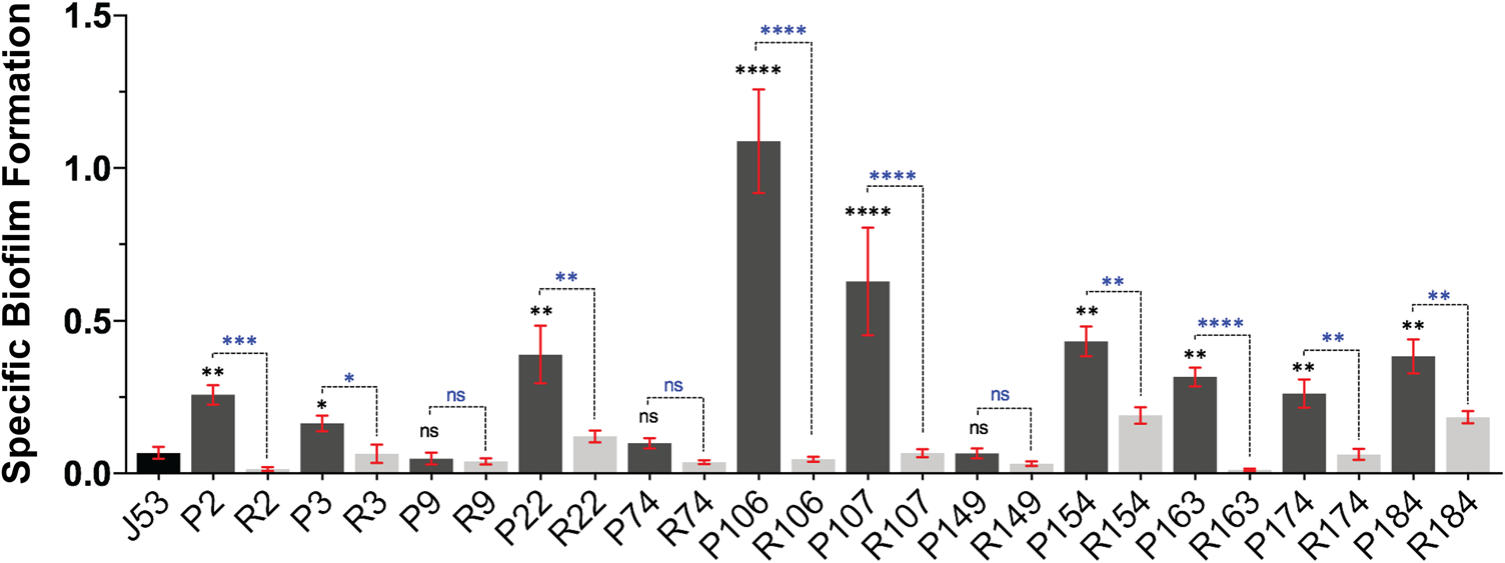
Biofilm formation efficiency of pathogenic *E. coli* strains, isolated from Ethiopian patients. Data was generated from a minimum of three biological and three technical replicates for every isolate and plotted using GraphPad-5.0. One-way ANOVA with inbuilt Tukey’s multiple comparison test was applied to calculate statistically significance between control strain *E. coli* J53 (black bar) and Parental strains (‘P’, dark grey bars) and respective recipient strains (‘R’, light grey bar). P < 0.0001: ***P < 0.001: **P < 0.01: *P > 0.05: non-significant (ns).

Knowledge of the interplay between bacterial biofilms and plasmids could benefit the development of therapeutic measures to control antimicrobial resistance plasmid transmissibility. Hence, we compared the ability of 12 donor parent strains and the corresponding plasmid recipient counterparts for their ability to form biofilms in a microtiter plate assay. The criteria for selecting this subset were: (1) a primary focus on the B2 phylotype because these are usually extra-intestinal bacteria, (2) a primary focus on the ST131 international high risk clone, (3) a spread of isolates having one to multiple plasmid replicon types, (4) a spread of isolates having one to multiple CTX-M types, and (5) all geographical study sites must be represented **[**NRL (National reference laboratory)—4; TASH (Tikur Anbessa Specialized hospital)—3; ARH (Ayder Referral Hospital)—3; JUH (Jimma University Hospital)—1] (Supplementary Table S1). Only two donor parental ‘P’ strains could be classified as a strong biofilm former—P106, or moderate biofilm former—P107 (Fig. 3). The remaining donor parental strains were either weak biofilm formers (P2, P3, P22, P154, P163, P174, and P184), or failed to form biofilms (P9, P74 and P149) under the experimental conditions tested (Fig. 3). Interestingly, only the plasmids transmissible from P22, P154 and P184 plasmid could confer to the recipient ‘R’ strains (R22, R154 and R184) the ability to form any degree of biofilm (Fig. 3). The replicon types identified in these three isolates were F-FIB, F-FIA-FIB and F-FIA-FIB, respectively (Supplementary Table S1). Hence, we could identify just three cases where the P22, P154 and P184 derived AMR plasmid(s) may have captured plasmid-encoded biofilm promoting factors.

### Influence of plasmid carriage on serum resistance

**Figure 4:**
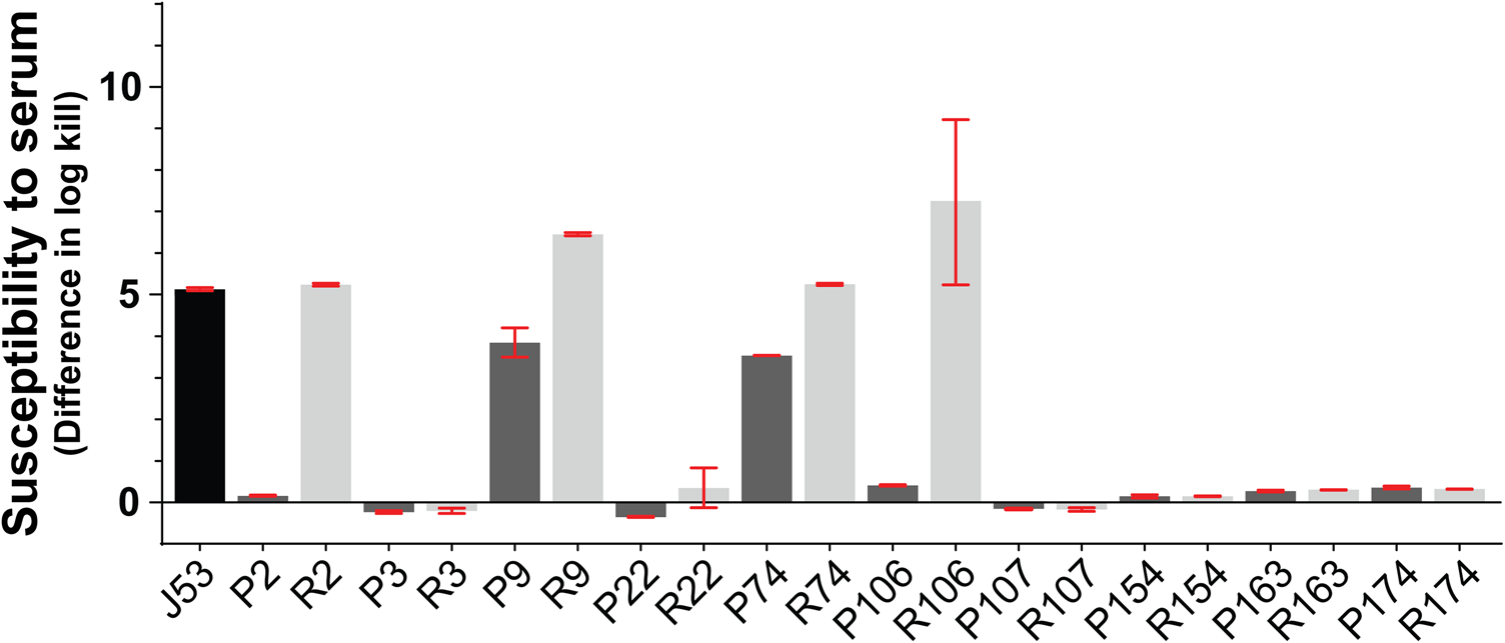
Survival efficiency of pathogenic *E. coli* strains grown in the presence of serum. Survival properties of 10 parental clinical isolates (dark grey bars) and their corresponding recipient strains (light grey bars) and the control strain J53 (black bar) after exposure to active human serum for 0 and 3 h. The susceptibility to killing was calculated as follows: log kill = (log10 CFU per milliliter of initially added bacteria—0 h)—(log10 CFU per milliliter of bacteria surviving the incubation after 3 h). GraphPad-5.0 was used to plot data from a minimum of two biological replicates of every isolate. Means and standard errors of the results are shown. One-way ANOVA with inbuilt Tukey’s multiple comparison test was applied to calculate statistical significance between the corresponding parental and recipient strains. P < 0.0001: ***P < 0.001: **P < 0.01: *P > 0.05: non-significant (ns).

Since carriage of AMR plasmids influence the extent of serum resistance^16^, we compared the ability of a selected group of donor parent strains and the corresponding plasmid recipient counterparts for their ability to confer resistance to normal human serum. Ten of the isolates selected above were also used in this serum sensitivity study (Supplementary Table S1). Two parental ‘P’ isolates (P9 and P74) and four recipient ‘R’ strains (R2, R9, R74 and R106) were totally sensitive to prolonged exposure to human serum (Fig. 4). This was comparable to the serum sensitive phenotype of the control strain *E. coli* J53. On the other hand, 8 parental strains (P2, P3, P22, P106, P107, P154, P163, and P174) and 6 recipient strains (R3, R22, R107, R154, R163, and R174) displayed extensive serum resistance. Hence, serum resistance in the strains P3, P22, P107, P154, P163, and P174 is influenced by transmissible AMR plasmid carriage. The replicon types identified in these six isolates were F-FIA-FIB-I1, F-FIA-FIB, F-FIA-FIB, F-FIA-FIB, W and F-FIA-FIB, respectively (Supplementary Table S1). In contrast, serum resistance in the strains P2, and P106 is not transmissible and must be associated with chromosomal encoded elements or elements encoded on non-transmissible plasmids.

## Discussion

This study is the first to report on plasmid transmissibility, replicon types and associated addiction systems among CTX-M-producing MDR *E. coli* clinical isolates from Ethiopia (Supplementary Table S1). It is also the first study from Ethiopia that describes these plasmid replicon types in association with the clonal distribution of CTX-M-producing *E. coli* clinical isolates. The data verify the long-held notion that horizontal gene transfer is a major contributor to the clonal expansion and widespread distribution of CTX-M-encoding genes in Ethiopia. These findings are alarming for it demonstrates that plasmid transmission is a major factor in the co-transfer of genes encoding resistance to non-cephalosporins antimicrobials among bacterial populations in Ethiopia. Consequently, in the absence of any intervention rapid spread of multiple drug resistance among bacterial populations in clinical, agricultural and community settings will continue unabated. An uneasy solace is for last resort carbapenem drugs, such as meropenem, where the isolates were still highly susceptible. Possible resistance to colistin, which is a last-resort treatment for MDR Gram-negative infections, was not tested because it was not approved for clinical use in Ethiopia at the time of the study.

While 75% of the isolates examined contained CTX-M genes on transmissible plasmids, the remaining 25% of isolates were unable to transfer these genes to the *E. coli* recipient. Moreover, the alternative β-lactamase *bla*_SHV_ was also non-transmissible. These non-transferable *bla*_CTX-M_ and *bla*_SHV_ genes are likely to be integrated into the host chromosome or be present on non-transmissible plasmids. These isolates are worthy of further genetic characterization as it may provide some new clues on the increased genetic heterogeneity among the markers for MDR and their dissemination. A precedent for this type of novel discovery is the chromosomal location of *bla*_CTX-M-15_ in the very extensive AMR and virulent subclone of ST131 H30Rx^20^. This origin was due to the mobilization of a plasmid-located Tn3-like IS*Ecp1*-*bla*_CTX-M-15-orf477_ element that subsequently integrated into the bacterial genome. IS*Ecp1* is an IS that contributes to the effective capture, expression, and mobilization of AMR genes from multiple sources, and is commonly located in the upstream of region of the CTX-M encoding genes^21,22^. Our observation of IS*Ecp1* in 97.3% of parental strains and 94.7% of the corresponding recipient strains corroborates these earlier findings.

Although exact genetic associations could not be directly determined without plasmid sequencing data, 59 (78.7%) transferrable *bla*_CTX-M_ genes appeared to be located on narrow-host-range IncF plasmids. This agrees with IncF plasmids being the major carrier of *bla*_CTX-M_ genes^23^. IncF plasmids encoding for CTX-Ms are detected in a range of *E. coli* sequence types^24^, but have a particularly strong association with the global spread of CTX-M-15 producing *E. coli* ST131^23^. IncF plasmids contribute various AMR determinants and virulence associated factors that create competitive fitness advantages that select for the success of the ST131 clone^25^, and the evolution of ST131 sub-lineages such as H30, which include H30R1 and H30Rx^7,25,26^. Consistent with this profile is our observation of IncF plasmids harboured by isolates associated with a variety of *E. coli* sequence type lineages, including the international high risk *E. coli* ST131 lineage. This indicates that the widespread prevalence of CTX-M-15 encoding genes in Ethiopia is facilitated by both host cell clonal expansions and horizontal gene transfer by IncF plasmids. We also identified IncF plasmids in *E. coli* phylogenetic groups primarily considered to be commensal *E. coli*, corroborating an earlier claim^27^.

We suspect that the IncF plasmids identified in this study have either single replicons or multiple replicons. The different combinations can reflect the fusion between different types creating a replicon chimera, or that multiple plasmids simultaneously coexist in the same cell^18,24,28,29^. This phenomenon is quite helpful to establish and trace relevant epidemiologic plasmid lineages. However, the fitness advantages conferred by a plasmid having multiple replicons within the same incompatibility group is not clear, since it surely would raise issues of replication coordination, regulation, and instability brought about by incompatibility phenomena. This contrasts with plasmids co-opting replicons from different incompatibility groups, which would extend replication opportunities within diverse hosts. Hence, follow-up work focused on whole genome or plasmid sequencing should assess if the IncF plasmid replicons represent discrete and intact genetic entities, or whether they are merely fusions between different types creating replicon chimeras, as has been previously reported^18^. Assessing replicon functionality is also warranted.

Interestingly, from eight addiction system types analysed in this study, we could detect three type I and three type II addiction systems. In the parental donor isolates this amounted to 337 combinations of addition systems. However, just 176 addiction system combinations were detected in the recipient transconjugants. As suggested by others, the implication of these findings is that addiction systems might be positioned on a non-conjugative plasmid or in the chromosome, and those located on conjugative plasmids are associated with the transmissible *bla*_CTX-M_ genes^19,30,31^. Moreover, almost all detected addiction systems in the recipient strains were carried on IncF plasmids, corroborating previous findings^32^. Plasmid addiction systems play important roles in plasmid stability and maintenance in a bacterial population^33^, and can enhance bacteria fitness under adverse environmental conditions^34^. Hence, our data indicate that IncF plasmids use multiple addiction systems for maintenance and stability during horizontal dissemination of *bla*_CTX-M_ genes within Ethiopian isolates.

An additional key element to this is the finding that these *bla*_CTX-M_ harbouring plasmids also possess the possibility to disseminate other resistant genes of clinical significance, including those that confer resistance to sulfamethoxazole/trimethoprim, ciprofloxacin, amoxicillin-clavulanic acid, gentamicin, amikacin, and cefoxitin. This highlights the potential of plasmids harbouring *bla*_CTX-M_ genes to quickly disseminate MDR in hospital, community, and agricultural settings. Thus, a better understanding of the origin and evolution of non-beta-lactam antibiotics resistance in Ethiopia is required.

Despite our identification and initial characterization of transmissible AMR plasmids in *E. coli* isolates collected at limited healthcare settings, it is evident that pivotal knowledge concerning the genetic diversity that might exist among them is still lacking. Follow up work should focus on further genetic characterization of the plasmids to better define the extent of their diversity, and to provide important information connecting plasmid backbone and replicon type with combinations of acquired multiple resistance genes and addiction modules as well as other phenotypic traits associated with pathogenicity such as serum resistance and the capability to form biofilms. Achieving this would require a method that combines S1-nuclease mediated cleavage of the plasmids, followed by pulsed field gel electrophoresis and Southern blotting^35^, and also in combination with direct plasmid sequencing, or even through whole genomic sequencing. Considering that most transmissible plasmids in this study were based on the IncF family, which in other studies has displayed extensive genetic diversity^36–39^ we speculate that this would be true also for plasmids harboured in our isolate collection. Moreover, a significant percentage of transmissible plasmids possessed a non-typed replicon according to our PBRT assay. Hence, applying the more discerning genetic methods on this group of isolates will also provide new clues concerning dissemination of AMR among various *E. coli* populations in Ethiopia.

Apart from the IncF plasmids, we were also interested in the isolates that harboured transmissible narrow-host-range plasmids with an IncI1-Iγ or IncY replicon. Although not confirmed by sequence-based methods, the IncI1-Iγ and IncY plasmids identified in our study were often found associated with the *bla*_CTX-M-15_ gene. Interestingly, plasmids with these replicons have been detected in bacteria isolated from animals produced for food and associate with various AMR genes^40–43^. Based on this precedent, it is possible that the IncI1-Iγ and IncY plasmids identified herein may have an animal origin, which could be a source for human infections in Ethiopia. This will be confirmed in future work with Ethiopian bacterial collections expanded to include isolates from wider community and agricultural sources. The only other replicon detected in our study was IncL/M. This is a broad-host-range replicon allowing for greater transmission among diverse bacterial species, as evidenced by an association between IncL/M plasmids and *bla*_CTX-M-3_ and *bla*_OXA-48_ dissemination^44,45^.

Various *E. coli* pathotypes possess a variety of virulence associated factors which support their entry, colonization, survival, and dissemination within and between infected human and animal hosts. Definitive conclusions concerning pathotypes of our isolates still require sequence analysis to identify the presence of hallmark virulence genes that have been defined in earlier studies^46–48^. It is well established that virulence associated factors can be encoded within mobile genetic elements, such as plasmids^49^. This is also reflected in this study that revealed survivability of a subset of parental isolates and their corresponding plasmid recipient strains in normal human serum, which is attributable to specific genes carried on the CTX-M-encoding resistance plasmids. Although the data is limited, it hints to the fact that AMR plasmids from many *E. coli* isolates sourced in Ethiopia likely also encode for other properties that can influence lifestyle choices important for environmental survival and host pathogenicity.

We also noted that most isolates containing the *bla*_CTX-M-14_, *bla*_CTX-M-15_ and *bla*_CTX-M-27_ genes distributed among the B2, D, and F phylo-groups. These likely represent extra-intestinal pathogenic *E. coli* (ExPEC) isolates, since global population studies routinely associate ExPEC bacteria within the B2 and D phylo-groups^50^. This is a serious concern given that ExPEC bacteria are a major global clinical problem^51^. Hence, we speculate a high prevalence of ExPEC in Ethiopia, although this needs to be verified on more extensive *E. coli* collections. This will be achievable because of our access to a diverse and expanding *E. coli* isolate collection via ongoing One Health laboratory-based AMR surveillance initiative in Ethiopia. The identification of virulence associated factors co-localising with plasmid-encoded AMR genes will have major ramifications for the evolution of novel and re-emerging bacterial pathogens that will pose acute health risk.

There is also interest in the isolates associated with the phylogenetic groups A, B1 and C which are unlikely to be ExPEC strains. Rather, they must be either intestinal non-pathogenic commensal isolates, or intestinal pathogenic isolates (InPEC). Either way, they have been isolated from non-stool samples, chiefly urine, suggesting an association with extra-intestinal infections, and which would require a translocation from the gastrointestinal tract. Since InPEC rarely caused extra-intestinal infections^52^, we suspect that the remaining extra-intestinal isolates belonging to the other phylogenetic groups A, B1 and C originated as commensal *E. coli*. This idea needs confirmation, but precedent comes from knowing that commensal *E. coli* strains can participate in extra-intestinal infections when the gastrointestinal barrier is breached especially in immune-compromised patients and when the bacterial load is particularly high^53^.

Our study has certain limitations. It did not directly determine which *bla*_CTX-M_ genes were genetically linked with the identified plasmid replicon types. Genetic characterization of the isolates that did not transfer *bla*_CTX-M_ genes was also not performed. Further, reliable genetic associations could not be directly inferred without plasmid sequencing data. Moreover, our identification and initial characterization of transmissible AMR plasmids in *E. coli* isolates were collected from limited healthcare settings and does not have nationwide representation. Thus, the results need confirmation in future work on collections expanded to include isolates from wider communities and agricultural sources. In future studies, the relationship between efficacy of plasmid transfer and the genetic features of the plasmids will be scrutinised, for this information will be relevant not only for epidemiologists and Ethiopian surveillance but also for the global scientific community to associate the success of the spread and dissemination of an antibiotic resistance gene with a plasmid type and mobility.

In conclusion, we report a high prevalence of IncF-like plasmids that might be involved in the mobilization of CTX-M and other AMR genes in Ethiopia. Mostly identified from the international successful ST131 lineage, these plasmids harbour the IS*Ecp1* element for effective gene capture as well as multiple addiction systems to select for plasmid maintenance in daughter cells. The data indicate the underlying molecular basis for the previously reported extensive prevalence of *bla*_CTX-M-15_ in Ethiopia^11^. Knowledge of the role of transmissible plasmids in the spread of AMR genes among extra-intestinal *E. coli* populations in Ethiopia is an important step. Not only will it help strengthen national infection prevention and control systems, but it also opens up the possibility of developing therapeutic strategies to target bacteria harbouring such plasmids to limit their subsequent acquisition and transmission of AMR within bacterial populations^54,55^. Overall, the data represent high AMR plasmid carriage among CTX-M ESBL-producing *E. coli* isolates from four facilities in Ethiopia. As a result, the potential for plasmid transmissibility is very high, as is the likelihood of further rapid spread of AMR genes of diverse families.

## Methods

### Study design and samples

The isolates were retrieved from a biobank after having been collected in 2018 from four geographically distinct facilities as part of ongoing national AMR surveillance initiative. The initiative was launched in 2017 by the EPHI under the supervision of the Ministry of Health and operates under the auspices of the *World Health Organization Global AMR and Use Surveillance* (*GLASS*) initiative (https://www.who.int/initiatives/glass). Detailed sample collection procedure including patient inclusion and exclusion criteria is reported elsewhere^11,56^ and strictly adhered to standard practices for clinical microbiological sampling established by the Ohio State University Global One Health initiative^57^. All in the biobank are clinical isolates and not isolates from the general population or other epidemiologic scenarios.

Initial phenotypic characterization, strain screening, phylo-typing as well as β-lactamase gene detection and antibiotic susceptibility testing were reported for 204 ESBLs-producing *E. coli* clinical isolates in our recent study^11^. In the current investigation, we considered 100 CTX-Ms-producing isolates obtained from urine (n = 84), pus (n = 10) and blood (n = 6) from the original set. It was sufficient to focus on just 100 isolates because all the CTX-Ms-encoding gene types identified in our initial study were included within this sub-collection. However, the over-representation of isolates from urine suggests that most of these strains will be enriched for virulence factors associated with genitourinary invasion. Moreover, we have no data on the antimicrobial exposure at the time of collection. This can be relevant because it is conceivable that certain patients from whom the isolates were obtained were receiving antimicrobial therapy, creating potential for bias toward over-representation of certain antimicrobial resistance genes.

### Ethics approval and consent to participate

The study was approved by the *EPHI Scientific and Ethical Review Board* (EPHI-IRB-054–2017) and the *College of Natural and Computational Sciences Institutional Review Board*, Addis Ababa University (CNS-IRB/039/2019). As all bacterial isolates included in this study were sourced from a biobank, the study did not directly involve patients, human material, or personal data identifiers.

### Plasmid transmissibility testing

Plasmids were transferred by either the conjugation or chemical transformation method. Conjugation was performed by a mating assay using *E coli* J53 AziR as recipient strain^58^. Trans-conjugants were selected on Luria-Bertani agar (LA) plates containing sodium azide (150 µg/mL) and cefotaxime (2 µg/mL). This necessitated that all donor strains were pre-tested for susceptibility to sodium azide and *E coli* J53 AziR was pre-tested for susceptibility to cefotaxime. If plasmids were non-conjugative, chemical transformation was performed using the recipient strain *E. coli* HB101 (Promega, Sweden). Plasmids were purified using the GeneJET plasmid miniprep kit (Thermo Fisher Scientific Inc.) and transformed into the chemically competent recipient strain using heat shock at 42 °C. Transformants were selected on LA plates supplemented with cefotaxime (2 µg/mL).

### ESBLs-gene detection and antibiotic susceptibility

Isolates were analysed for the presence of ESBL-encoding genes *bla*_TEM,_ *bla*_SHV,_ *bla*_CTX-M_ and *bla*_OXA_ using a combination of established PCR and sequencing methods as previously described^59^. Purified PCR products were sequenced using the service of Eurofins genomics (Ebersberg, Germany). The β-lactamase gene types were identified by alignment with sequences in GenBank using BLAST (http://www.ncbi.nlm.nih.gov/BLAST). Antibiotic susceptibility testing was done for 10 antibiotics against transferable parental isolates and their corresponding recipients of AMR plasmids to confirm the transfer of *bla*_CTX-M_ and to detect any associated transfer of other resistance phenotypes. The assay used the disk-diffusion method on Mueller–Hinton agar plates following CLSI recommendations. The panel of antibiotic containing disks (Oxoid LTD, Basingstoke, Hampshire, England) along with their abbreviated names were amoxicillin-clavulanic acid (AMC-20/10 µg), cefoxitin (FOX-30 µg), cefotaxime (CTX-30 µg), ceftazidime (CAZ-30 µg), cefepime (CEF-30 µg), gentamicin (GEN-10 µg), amikacin (AMK-30 µg), ciprofloxacin (CIP-5 µg), meropenem (MEM-10 µg) and sulfamethoxazole/trimethoprim (SXT-23.75/1.25 µg). Susceptibility was interpreted according to CLSI document M100-S30 (CLSI, 2020). ESBL-negative *E. coli* ATCC 25,922 and ESBL-positive *K. pneumoniae* subsp. pneumoniae ATCC 700,603 (Microbiologics Inc., Saint Cloud, Minnesota, USA) were used as reference strains.

### AMR plasmid replicon typing

A PBRT protocol involving 18 primer pairs in 5 multiplex- and 3 simplex-reaction setups^60^ was used to identify AMR plasmid replicon types FIA, FIB, FIC, HI1, HI2, I1-Ig, L/M, N, P, W, T, A/C, K, B/O, X, Y, F, and FIIA.

### Addiction system detection

Eight addiction systems were determined for the 75 parental donors and their respective recipient strains using previously described PCR primers pairs and amplification conditions^19^. These included the detection of three type I addiction systems [Hok-Sok (*hok-sok*) (host-killing), PndA-PndC (*pndAC*) (promotion of nucleic acid degradation), and SrnB-SrnC (*srnBC*) (stable RNA negative)] and five type II addiction systems [PemK-PemI (p*emKI*) (plasmid emergency maintenance), CcdA-CcdB (*ccdAB)* (coupled to cell division), RelB-RelE (*relBE*) (relaxed control stable RNA synthesis), ParD-ParE (*parDE*) (DNA replication), and VagC-VagD (*vagCD*) (virulence associated proteins)].

### PCR-based detection of ISEcp1 element

Detection of the insertion sequence IS*Ecp1* was determined by PCR using the combination of IS*Ecp*1 primer and CTX-M reverse consensus primer (MA1 reverse) as previously described^61^. An amplified product is indicative of the IS*Ecp1* element situated upstream of the *bla*_CTX-M_ genes. The PCR products were purified and confirmed by sequencing.

### Plasmid transmissibility and biofilm formation

Biofilm formation was determined for 12 parental isolates. The isolates chosen and their detected genotypes and phenotypes are listed in Supplementary Table S1. All parental isolates belonged to the known extra-intestinal pathogenic *E. coli* (phylogenetic group B2 and D). All except isolate 149 are the international high-risk clone ST131. All produce at least one CTX-M type, and all except isolate 74 exhibited the detection of more than one IncF plasmid replicon type. The measurement of biofilm forming capacity of the parental isolates and their corresponding recipients used a previously described protocol with slight modification^62^. Briefly, both groups of bacteria were grown in M63 minimal media with glycerol as the carbon source, and with respective antibiotics overnight at 37 °C with aeration. Cefotaxime (2 µg/ml) was used for donor parental strains and 2 µg/ml cefotaxime and 100 µg/ml sodium azide were used for recipients. The strain *E. coli* J53 AziR was used as a control and grown in the M63 medium containing 100 µg/ml sodium azide. From overnight bacterial cultures, 3 µl aliquots were mixed with in 147 µl fresh M63 medium in the wells of a sterile 96-well round-bottom µl dish and incubated overnight at 37 °C. Developed biofilms were heat-fixed and stained with 0.1% w/v crystal violet solution. The stained biomass was recovered by solubilisation in 33% (v/v) glacial acetic acid. The extent of solubilized biofilm was then recorded spectroscopically at an absorbance of 560 nm, and the efficiency of biofilm formation calculated by normalization with planktonic growth recorded as the optical density at a wavelength of 600 nm. The specific biofilm formation (SBF) was determined using the formula SBF = (AB-CW)/G, where AB is OD_560_ of stained cells, CW is OD_560_ of control wells cultured with M63 medium only, and G is the OD_600_ of the bacteria growth calculated from G = OD_600_ (24 h)—OD_600_ (0 h). The strains were categorized as *weak biofilm former* (SBF ≤ 0.5), *moderate biofilm former* (SBF = 0.5–1.0) and *strong biofilm former* (SBF ≥ 1.0). For logistical reasons, the sample size was restricted to 12 to enable a minimum of three biological replicates with three technical repeats to ensure data quality. Furthermore, the selection process ensured that the parental isolates represented the different phylogenetic backgrounds.

### Serum resistance measurements

Serum sensitivity was determined for 10 parental isolates and their corresponding trans-conjugants using a previously described protocol^49^. Briefly, the strains were grown in LB broth overnight at 37 $^\circ$C with aeration. Five µl of the overnight cultures were sub-cultured into 495 µl fresh LB broth and grown statically for 2 h at 37 $^\circ$C. Following centrifugation at 7600 g for 3 min, pellets were re-suspended in 500 µl phosphate-buffered saline. Volumes of 20 µl from the washed bacteria were mixed with 180 µl of normal human serum in a 96-well flat bottom microtiter dish and incubated statically at 37 °C for 3 h. At 0 h and 3 h time points, 20 µl was removed from the wells and plated after suitable serial dilution on LB plates containing 2 µg/ml cefotaxime for parental strains, 2 µg/ml cefotaxime and 100 µg/ml sodium azide for trans-conjugants, and 100 µg/ml sodium azide for the *E. coli* J53 AziR control. The number of colony forming units (CFUs) of bacteria was determined after the plates were incubated overnight at 37 °C. Susceptibility to active serum was calculated as follows: log kill = (log10 CFU/µl of initially added bacteria—0 h)—(log10 CFU/µl of bacteria surviving the incubation after 3 h) according to a previous report^63^. All experiments were conducted in duplicate. The selection process of the 10 isolates ensured that the parental isolates represented the different phylogenetic backgrounds. The recipient J53 strain alone was used as a control in all assays.

### Statistical analysis

The data was prepared using Excel spread sheets (Microsoft Office) and imported to SPSS version 20.0. The frequencies of different variables were calculated. Cross-tabulation and graphs were used to present the different relation between data.

## Data Availability

The datasets used and/or analysed during the current study are available from the corresponding author upon reasonable request.

## Supplementary Table

Supplementary Information.

## Abbreviations

AMR: Antimicrobial resistance
ASLM: African Society for Laboratory Medicine
AST: Antimicrobial susceptibility test
*Bla*: β- Lactamase coding gene
BLAST: Basic Local Alignment Tool
CFU: Colony forming unit
CLSI: Clinical and Laboratory Standard Institution
CTX-M: Active on Cefotaxime, First Isolated at Munich
ENAO: Ethiopian National Accreditation Office
ESBL: Extended spectrum β-lactamases
HG: Horizontal gene transfer
IRB: Institutional Review Board
ID: Identification
Inc: Incompatibility
IS: Insertion sequence
ISO: International Organization for Standardization
LA: Lauria-Bertani Agar
LB: Lauria-Bertani
MLST: Multilocus sequence typing
OXA: Oxacillinase
SBF: Specific biofilm formation
SHV: β-Lactamase enzyme named after sulphydryl variable
SLIPTA: The stepwise laboratory quality improvement process towards accreditation
ST: Sequencing type
TEM: β-Lactamase enzyme named after a Greek patient Temoniera
